# Evidências Brasileiras Reforçam a Aplicabilidade dos Escores H2FPEF e HFA-PEFF na ICFEp

**DOI:** 10.36660/abc.20250361

**Published:** 2025-08-12

**Authors:** Pedro Silvio Farsky

**Affiliations:** 1 Instituto Dante Pazzanese de Cardiologia São Paulo SP Brasil Instituto Dante Pazzanese de Cardiologia, São Paulo, SP – Brasil; 2 Hospital Israelita Albert Einstein São Paulo SP Brasil Hospital Israelita Albert Einstein, São Paulo, SP – Brasil

**Keywords:** Medição de Risco, Prognóstico, Insuficiência Cardíaca Diastólica

A insuficiência cardíaca com fração de ejeção preservada (ICFEp) continua sendo um desafio diagnóstico na cardiologia moderna. Representando aproximadamente metade de todos os casos de insuficiência cardíaca, essa condição apresenta fisiopatologia complexa, critérios diagnósticos frequentemente subjetivos e opções terapêuticas mais limitadas.^1,2^ Nesse contexto, o uso de sistemas de pontuação diagnóstica, como H2FPEF e HFA-PEFF, ganhou importância, fornecendo aos médicos ferramentas padronizadas e baseadas em evidências para orientar tanto o diagnóstico quanto a estratificação de risco.^2,3^

Um estudo prospectivo realizado em um hospital terciário brasileiro fornece dados relevantes sobre a aplicabilidade desses escores em nossa população.^4^ Realizado entre março de 2019 e dezembro de 2021, o estudo incluiu 103 pacientes com diagnóstico de ICFEp, acompanhados por aproximadamente 2,4 anos. Além da caracterização clínica e ecocardiográfica, todos os pacientes foram submetidos a teste de esforço, e os escores H2FPEF e HFA-PEFF foram calculados. Os pacientes foram então categorizados em grupos de probabilidade intermediária e alta, de acordo com os pontos obtidos, e acompanhados para o desfecho composto de mortalidade por todas as causas e hospitalizações por insuficiência cardíaca.

Os resultados são estimulantes e têm implicações clínicas práticas. O escore H2FPEF demonstrou melhor desempenho na previsão de desfechos adversos, com uma área sob a curva ROC (*Receiver Operating Characteristic*) de 0,637 (p = 0,035). Em contraste, o escore HFA-PEFF apresentou uma AUC de 0,572, sem significância estatística (p = 0,270).^4^ Além disso, a análise de Kaplan-Meier revelou que pacientes com alta probabilidade de ICFEp, de acordo com ambas as pontuações, tiveram significativamente mais eventos cardiovasculares quando comparados àqueles com pontuações intermediárias ou discordantes.

Esses achados oferecem três contribuições importantes para a prática cardiológica brasileira.

Em primeiro lugar, reforçam a utilidade prognóstica dos escores H2FPEF e HFA-PEFF. Embora ambos tenham sido desenvolvidos principalmente para fins diagnósticos — H2FPEF nos Estados Unidos, com base em variáveis clínicas simples como IMC, idade, fibrilação atrial, hipertensão, pressão pulmonar e volume atrial esquerdo;^2,3^ e HFA-PEFF pela Sociedade Europeia de Cardiologia, com uma abordagem mais estruturada e gradual, incluindo ecocardiografia, biomarcadores e testes funcionais — ambos demonstraram, em vários estudos internacionais, uma associação com resultados clínicos.^5^ Este estudo brasileiro confirma que tal associação também é válida em nossa população, historicamente sub-representada na literatura internacional.

Em segundo lugar, os resultados sugerem que o escore H2FPEF pode ser uma ferramenta mais sensível para estratificação de risco em pacientes com ICFEp. Sua simplicidade, baseada em variáveis clínicas facilmente obtidas em ambientes ambulatoriais, o torna particularmente atraente no contexto brasileiro, onde o acesso a testes diagnósticos mais complexos pode ser limitado.^6^ Embora a pontuação HFA-PEFF ofereça robustez metodológica, sua aplicação completa muitas vezes requer infraestrutura de diagnóstico que não está disponível fora dos principais centros.

Em terceiro lugar, a observação de que pacientes com alta probabilidade de acordo com ambas as pontuações apresentaram mais eventos, enquanto aqueles com pontuações intermediárias ou discordantes tiveram um curso mais favorável, levanta a possibilidade de uma estratégia combinada no uso do escore. Em outras palavras, o uso de ambas as ferramentas de forma complementar para identificar pacientes cujo risco é consistente em ambos os modelos e, portanto, pode justificar um monitoramento mais rigoroso e potencial intensificação terapêutica.

No entanto, o estudo apresenta algumas limitações que devem ser consideradas. O tamanho relativamente pequeno da amostra e o fato de ter sido conduzido em um único centro limitam a generalização dos resultados. Além disso, o número de eventos foi modesto, o que pode ter impactado a precisão estatística, particularmente para o escore HFA-PEFF. No entanto, esses dados lançam luz sobre um tópico clínico altamente relevante e destacam a necessidade de estudos multicêntricos maiores no Brasil.

No contexto da prática clínica, este estudo fornece subsídios para a incorporação do escore H2FPEF como uma ferramenta útil tanto para diagnóstico quanto para prognóstico em pacientes com insuficiência cardíaca suspeita ou confirmada com fração de ejeção preservada (ICFEp). Seu uso pode auxiliar nos processos de tomada de decisão, como a necessidade de investigação adicional, escalonamento terapêutico ou acompanhamento mais próximo. Embora o escore HFA-PEFF não tenha demonstrado superioridade prognóstica nesta coorte, ele permanece valioso como uma abordagem estruturada, particularmente em ambientes acadêmicos ou quando a incerteza diagnóstica persiste após a avaliação inicial.^6^

Por fim, é essencial enfatizar a importância deste estudo para o fornecimento de evidências nacionais em um campo tradicionalmente dominado por dados internacionais. A ICFEp é uma condição prevalente, impactante e desafiadora. Escores como H2FPEF e HFA-PEFF não substituem o julgamento clínico, mas são aliados valiosos como ferramentas diagnósticas, auxiliando no processo de estratificação de risco. À medida que avançamos em direção a uma medicina cada vez mais personalizada, baseada em evidências e sensível ao contexto local, estudos como este são essenciais para orientar a tomada de decisões à beira do leito.
