# Supplementary material for: Evidências Brasileiras Reforçam a Aplicabilidade dos Escores H2FPEF e HFA-PEFF na ICFEp
Source: Arq Bras Cardiol. 2025 Aug 12;122(6):e20250361. [Article in Portuguese] doi: 10.36660/abc.20250361 (PMC13718330; doi:10.36660/abc.20250361)
Supplement: Translation

# Translation: Brazilian Evidence Reinforces the Applicability of the H2FPEF and HFA-PEFF Scores in HFpEF

**DOI:** 10.36660/abc.20250361i

**Authors:** Pedro Silvio Farsky

**Affiliations:** 1 São Paulo SP Brazil Instituto Dante Pazzanese de Cardiologia, São Paulo, SP – Brazil; 2 São Paulo SP Brazil Hospital Israelita Albert Einstein, São Paulo, SP – Brazil

**Keywords:** Risk Assessment, Prognosis, Diastolic Heart Failure

Heart failure with preserved ejection fraction (HFpEF) remains a diagnostic challenge in modern cardiology. Representing approximately half of all heart failure cases, this condition presents with complex pathophysiology, often subjective diagnostic criteria, and more limited therapeutic options.^1,2^ In this context, the use of diagnostic scoring systems, such as H2FPEF and HFA-PEFF, has gained importance, providing clinicians with standardized and evidence-based tools to guide both diagnosis and risk stratification.^2,3^

A prospective study conducted at a Brazilian tertiary hospital provides relevant data on the applicability of these scores in our population.^4^ Conducted between March 2019 and December 2021, the study included 103 patients diagnosed with HFpEF, who were followed for approximately 2.4 years. In addition to clinical and echocardiographic characterization, all patients underwent exercise testing, and both the H2FPEF and HFA-PEFF scores were calculated. Patients were then categorized into intermediate- and high-probability groups according to the score points obtained and followed for the composite outcome of all-cause mortality and heart failure hospitalizations.

The results are stimulating and have practical clinical implications. The H2FPEF score demonstrated better performance in predicting adverse outcomes, with an area under the receiver operating characteristic curve (AUC) of 0.637 (p = 0.035). In contrast, the HFA-PEFF score presented an AUC of 0.572 without statistical significance (p = 0.270).^4^ Moreover, Kaplan-Meier analysis revealed that patients with a high probability of HFpEF, according to both scores, had significantly more cardiovascular events when compared to those with intermediate or discordant scores.

These findings offer three important contributions to Brazilian cardiology practice.

First, they reinforce the prognostic utility of the H2FPEF and HFA-PEFF scores. Although both were developed primarily for diagnostic purposes — H2FPEF in the United States, based on simple clinical variables such as BMI, age, atrial fibrillation, hypertension, pulmonary pressure, and left atrial volume;^2,3^ and HFA-PEFF by the European Society of Cardiology, with a more structured and stepwise approach including echocardiography, biomarkers, and functional testing — both have shown, in various international studies, an association with clinical outcomes.^5^ This Brazilian study confirms that such an association is also valid in our population, which has historically been underrepresented in the international literature.

Second, the results suggest that the H2FPEF score may be a more sensitive tool for risk stratification in patients with HFpEF. Its simplicity, based on easily obtainable clinical variables in outpatient settings, makes it particularly attractive in the Brazilian context, where access to more complex diagnostic tests may be limited.^6^ Although the HFA-PEFF score offers methodological robustness, its full application often requires diagnostic infrastructure that is unavailable outside major centers.

Third, the observation that patients with a high probability according to both scores experienced more events, while those with intermediate or discordant scores had a more favorable course, raises the possibility of a combined strategy in score usage. In other words, using both tools in a complementary fashion to identify patients whose risk is consistent across both models and, therefore, may warrant closer monitoring and potential therapeutic intensification.

However, the study has some limitations that must be considered. The relatively small sample size and the fact that it was conducted in a single center limit the generalizability of the results. Additionally, the number of events was modest, which may have impacted statistical accuracy, particularly for the HFA-PEFF score. Nonetheless, these data shed light on a highly relevant clinical topic and highlight the need for larger multicenter studies in Brazil.

In the context of clinical practice, this study provides support for incorporating the H2FPEF score as a useful tool for both diagnosis and prognosis in patients with suspected or confirmed heart failure with preserved ejection fraction (HFpEF). Its use may assist in decision-making processes, such as the need for further investigation, therapeutic escalation, or closer follow-up. Although the HFA-PEFF score did not demonstrate prognostic superiority in this cohort, it remains valuable as a structured approach, particularly in academic settings or when diagnostic uncertainty persists after the initial evaluation.^6^

Finally, it is essential to emphasize the significance of this study in providing national evidence in a field traditionally dominated by international data. HFpEF is a prevalent, impactful, and challenging condition. Scores such as H2FPEF and HFA-PEFF do not replace clinical judgment, but they are valuable allies as diagnostic tools, aiding in the risk stratification process. As we move toward increasingly personalized, evidence-based medicine that is sensitive to the local context, studies like this are essential for guiding bedside decision-making.
